# Characterizing right-angled vessel in macular telangiectasia type 2 with structural optical coherence tomography

**DOI:** 10.1038/s41598-021-96789-4

**Published:** 2021-08-25

**Authors:** Yoo-Ri Chung, Young Ho Kim, Jaeryung Oh, Seong-Woo Kim, Christopher Seungkyu Lee, Cheolmin Yun, Boram Lee, So Min Ahn, Eun Young Choi, Sungmin Jang, Kihwang Lee

**Affiliations:** 1 Department of Ophthalmology, Ajou University School of Medicine, 164 World Cup-ro, Yeongtong-gu, Suwon, 16499 Korea; 2 Department of Ophthalmology, Korea University College of Medicine, Seoul, Korea; 3 Department of Ophthalmology, Yonsei University College of Medicine, Seoul, Korea; 4 Institute of Vision Research, Yonsei University College of Medicine, Seoul, Korea; 5 Institute of Human Barrier Research, Yonsei University College of Medicine, Seoul, Korea; 6 Retina Center, Saevit Eye Hospital, Goyang, Korea

**Keywords:** Diseases, Risk factors

## Abstract

We investigated the structural findings on spectral-domain optical coherence tomography (SD-OCT) related to the presence of right-angled vessels (RAV) in patients with macular telangiectasia (MacTel) type 2 with severity 3 in Korea. A retrospective multicenter cross-sectional study was conducted in six tertiary hospitals in Korea; the study included 116 MacTel type 2 eyes with severity 3. The SD-OCT findings were compared between eyes with RAV on fundus photography or fluorescein angiography and those without RAV. Logistic regression was performed to determine factors associated with the presence of RAV. Fifty eyes presented with RAV and 61 eyes without RAV. More eyes presented with only inner retinal (IR) cavities on SD-OCT among eyes without RAV than among those with RAV (*P* < 0.001). However, eyes with RAV presented with IR disorganization, outer retinal (OR) cavity, and ellipsoid zone (EZ) disruption more frequently than eyes without RAV did (all *P* < 0.001). These SD-OCT findings were significantly associated with the presence of RAV. The presence of RAV was closely related to IR disorganization, OR cavities, and EZ disruption on SD-OCT. These findings suggest an advanced phase of MacTel type 2.

## Introduction

The introduction of spectral domain optical coherence tomography (SD-OCT) has allowed detailed observation of the structural characteristics, such as cavities in the inner and outer retina, disruption of the ellipsoid zone (EZ), and retinal atrophy, of macular telangiectasia (MacTel) type 2^1,2^. However, the pathogenesis of MacTel type 2 has not been well described thus far. Recent studies indicate that MacTel type 2 is a result of degeneration or loss of Müller and photoreceptor cells with vascular changes as secondary manifestations^3^.

Unlike Gass’s proposal, Spaide et al.^4^ reported that the presence of right-angled veissels (RAV) was associated with retinal-choroidal anastomoses (RCA) without evidence of neovascularization, such as hemorrhage, exudation, and lipids on optical coherence tomography angiography (OCTA). These vascular connections between the retinal deep capillary plexus and choroid were not a late stage phenomenon, as proposed by Gass, but could be seen with RAV, which is considered a signature of stage 3^4^.

The previous studies based on OCTA reported that RCA were closely associated with the presence of RAV, showing RAV were seen in all eyes with RCA^4,5^. Although the relationship between EZ defect, outer retinal hyperreflective lesions and RAV was investigated by previous studies^4,5^, other structural findings on SD-OCT were not fully investigated. We previously reported the SD-OCT findings of MacTel type 2 in Korean patients; these patients presented with hyporeflective cavities and disruption of the external limiting membrane (ELM), EZ, and interdigitation zone (IDZ) as common features^6^. In this report, we investigated further various structural findings on SD-OCT related to the presence of RAV in patients with MacTel type 2 with severity 3.

## Results

Among patients with MacTel type 2, 111 eyes in 69 patients with severity 3 were included in this analysis. There was no difference in the mean age of patients with and without RAV, and patients were predominantly female regardless of the presence of RAV (Table 1). Best-corrected visual acuity (BCVA) was significantly better in eyes without RAV than in those with RAV.

**Table 1:** Baseline characteristics of included patients according to the presence of right-angled vessel.

	RAV	No RAV	*P* value
No. of patients	30	39	
Age, mean ± SD	65.5 ± 10.7	67.3 ± 10.0	0.471
Female, no. (%)	20 (67%)	30 (77%)	0.344
No. of eyes	50	61	
logMAR BCVA, mean ± SD	0.30 ± 0.26	0.21 ± 0.22	0.024*

*BCVA* best-corrected visual acuity, *RAV* right-angled vessel, *SD* standard deviation.

*Mean logMAR visual acuity was estimated by using generalized linear models and the generalized estimation equation method to account for the correlation between eyes. *P* value < 0.05 by generalized linear models and the generalized estimation equation method comparing the presence and absence of right-angled vessel.

**Figure 1:**
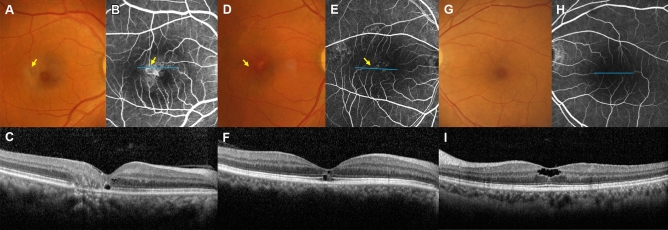
Representative cases of MacTel type 2 with severity 3. (**A**,**B**) A 58-year-old woman presented with a right-angled vessel (RAV, yellow arrows) on fundus photography (FP) and fluorescein angiography (FAG) of the right eye. (**C**) In the cross-section optical coherence tomography (OCT) B-scan (corresponding blue line in **B**), there were hyporeflective retinal cavities, ellipsoid zone (EZ) disruption, and interdigitation zone (IDZ) disruption. (**D**,**E**) A 59-year-old man with RAV on FP and FAG of the right eye. (**F**) Hyporeflective retinal cavities, EZ disruption, and IDZ disruption were noted on cross-section OCT B-scan (corresponding blue line in **E**). (**G**,**H**) A 59-year-old female without RAV on FP and FAG of the left eye. (**I**) In the cross-section OCT B-scan (corresponding blue line in **H**), there were only inner hyporeflective retinal cavities.

The presence of most SD-OCT findings was statistically different between the groups with and without RAV (Table 2). Fifty eyes presented with only inner retinal hyporeflective cavities, and this finding was more frequent in eyes without RAV than in eyes with RAV (64% vs. 22%, *P* < 0.001). In contrast, inner retinal disorganization was more frequent in eyes with RAV than in those without RAV (40% vs. 7%, *P* < 0.001). The following SD-OCT findings of the outer retina were also more frequent in eyes with RAV than in eyes without RAV: outer retinal hyporeflective cavities (52% vs. 23%, *P* = 0.002), ELM disruption (50% vs. 18%, *P* < 0.001), EZ disruption (78% vs. 28%, *P* < 0.001), IDZ disruption (84% vs. 34%, *P* < 0.001), and collapsed outer nuclear layer (28% vs. 5%, *P* = 0.001). Representative cases are presented in Fig. 1.

**Table 2:** SD-OCT findings of included eyes according to the presence of right-angled vessel.

	RAV (N = 50)	No RAV (N = 61)	*P* value
Disorganization of inner retina	20 (40%)	4 (7%)	< 0.001*
**Inner retinal cavity**	43 (86%)	58 (95%)	0.096
Only inner retinal cavity	11 (22%)	39 (64%)	< 0.001*
**Outer retinal cavity**	26 (52%)	14 (23%)	0.002*
Temporal	18 (36%)	9 (15%)	0.009*
Center	17 (34%)	8 (13%)	0.009*
Nasal	3 (6%)	5 (8%)	0.656
Above ELM	24 (48%)	14 (23%)	0.006*
Below ELM	26 (52%)	10 (16%)	< 0.001*
ELM disruption	25 (50%)	11 (18%)	< 0.001*
EZ disruption	39 (78%)	17 (28%)	< 0.001*
IDZ disruption	42 (84%)	21 (34%)	< 0.001*
ONL collapse	14 (28%)	3 (5%)	0.001*

*ELM* external limiting membrane, *EZ* ellipsoid zone, *IDZ* interdigitation zone, *ONL* outer nuclear layer, *RAV* right-angled vessel.

*P values < 0.05 by chi-square test or Fisher’s exact test.

We performed the logistic regression analysis to identify SD-OCT findings associated with the presence of RAV (Table 3). The following findings were associated with RAV: disorganization of the inner retina, outer retinal cavity, ELM disruption, EZ disruption, IDZ disruption, and collapsed outer nuclear layer. Among these factors, disorganization of the inner retina (odd ratio (OR) 4.358, 95% confidence interval (CI) 1.223–15.525, *P* = 0.023), outer retinal hyporeflective cavity (OR 2.690, 95% CI 1.049–6.899, *P* = 0.040), and EZ disruption (OR 5.346, 95% CI 2.069–13.815, *P* = 0.001) remained significant in the multinomial logistic regression analysis.

**Table 3:** Logistic regression for associated factors to the presence of right-angled vessel.

	OR	95% CI	*P* value
Age	0.178	0.937–1.012	0.974
Sex, male	1.902	0.815–4.438	0.137
Disorganization of inner retina	9.500	2.975–30.331	< 0.001*
Inner retinal cavity	0.318	0.078–1.300	0.111
Outer retinal cavity	3.637	1.610–8.215	0.002*
ELM disruption	4.545	1.930–10.703	0.001*
EZ disruption	9.146	3.835–21.955	< 0.001*
IDZ disruption	10.000	3.976–25.150	< 0.001*
ONL collapse	7.519	2.020–27.990	0.003*

*ELM* external limiting membrane, *EZ* ellipsoid zone, *IDZ* interdigitation zone, *ONL* outer nuclear layer.

*P values < 0.05 by logistic regression analysis.

## Discussion

In this analysis of MacTel type 2 eyes with severity 3, inner retinal disorganization, outer retinal hyporeflective cavity, and EZ disruption on SD-OCT were associated with RAV. These OCT findings have already been reported to be associated with visual deterioration in macular diseases such as diabetic macular edema and epiretinal membrane. In diabetic macular edema, inner retinal disorganization and EZ disruption were associated with increased disease severity and decreased vision^7^. Similarly, EZ attenuation was associated with decreased vision in eyes with epiretinal membrane^8,9^. These OCT findings were also closely related to visual acuity in MacTel type 2, suggesting that these findings could be used as markers of visual disturbance^6,10^. However, inner retinal hyporeflective cavities without outer retinal abnormalities, the presence of which has been reported at an early stage of the disease^6^, did not show a significant association with RAV in this study.

Recently, RCA, vessels connecting the deep vascular plexus and the choriocapillaris, have been reported in MacTel type 2 on OCTA^4,5,11^. Interestingly, these findings did not accompany any subretinal neovascularization seen in Gass stage 5^4^. Eyes with RCA presented with the following findings: hyperpigmentation, RAV, outer retinal hyperreflective lesion, and EZ defect on OCTA^4,5^. It is important to note that RCA were reported in two-thirds of MacTel type 2 cases, but this was not noted in eyes without RAV^4^.

In this analysis of MacTel type 2 eyes with severity 3, inner retinal disorganization, outer retinal hyporeflective cavity, and EZ disruption were SD-OCT findings associated with RAV. The association of RAV and RCA were previously reported^4,5^. Taken together, this suggests that the presence of these SD-OCT findings, which were related to RAV, might also be associated with RCA.

Powner et al.^3,12^ investigated the postmortem histology of patients with MacTel type 2 and reported a dramatic loss of Müller cells in the central retina. Hyporeflective cavities on OCT revealed no internal structures on histology, suggesting these cavities are filled with fluid^3^. Müller cells are responsible for the maintenance of the ion and water homeostasis of the retinal tissue and are also involved in the control of angiogenesis and neuronal signaling^13^. In a healthy retina, Müller cells dehydrate the retinal tissue via K^+^ channels and aquaporin-4 water channels on their plasma membrane and Kir4.1 channels on the perivascular processes of Müller cells^13^. However, in an ischemic or inflamed retina, the expression of the above-mentioned channels is dysregulated, resulting in Müller cell dysfunction^13^. Therefore, dysfunction of Müller cells may lead to surrounding cavities with fluid in MacTel type 2. Longstanding outer retinal cavities with atrophy of Müller cells, not by Müller cone cells, might cause the collapse of the retina and loss of photoreceptor cells, which would present as inner retinal disorganization and EZ disruption on SD-OCT. Moreover, Müller cells produce factors, such as pigment epithelium-derived growth factor (PEDF), tumor necrosis factor, and vascular endothelial growth factor (VEGF), that regulate the tightness of the endothelial barrier^13^. In diseased retinae, such as those in diabetic retinopathy, the production of VEGF is increased, while that of PEDF is reduced^13^. Hence, decreased release of anti-angiogenic factors, such as PEDF, may be associated with RAV (and RCA) in MacTel type 2.

Several limitations, including its retrospective nature, exist in this study. The lack of longitudinal evaluation is one of the limitations of this study, which is needed to investigate the progression of diseases and pathophysiology process of RAV and possibly RCA. Recently, several studies on longitudinal basis reported that the development of RCA might be associated with retinal subsidence and outer retinal hyperreflectivity^12,13^.

In conclusion, the present study showed that IR disorganization, OR cavity, and EZ disruption on SD-OCT were associated with the presence of RAV in MacTel type 2. This suggests that the presence of these SD-OCT findings, related with RAV, should be considered to have advanced phase MacTel type 2 in terms of vision with possible RCA.

## Methods

This study complied with the tenets of the Declaration of Helsinki, and the need for informed consent was waived by the Institutional Review Board (IRB) of each participating hospital given the retrospective nature of the study. The following six hospitals participated in this study, and the IRBs of each participating hospital approved this study: Ajou University Hospital (Suwon, Korea, IRB No: AJIRB-MED-OBS-18-222), Korea University Anam Hospital (Seoul, Korea, IRB No: 2018AN0327), Korea University Guro Hospital (Seoul, Korea, IRB No: 2018GR0344), Korea University Ansan Hospital (Ansan, Korea, IRB No: 2018AS0224), Severance Hospital (Seoul, Korea, IRB No: 3-2018-0221), and Gangnam Severance Hospital (Seoul, Korea, IRB No: 3-2018-0221).

We reviewed the medical records and SD-OCT findings of 111 patients diagnosed with MacTel type 2 with severity 3 from the datasets of the six hospitals from January 2009 to May 2019. Severity 3 was defined by the MacTel Study Group as moderate-to-marked foveal hyperautofluorescence with angiographic abnormalities and foveal atrophy documented on OCT^14,15^. BCVA and demographic data, including sex and age, were collected from medical records at the time of diagnosis or first visit. The diagnosis of MacTel Type 2 was made by an ophthalmologist for each patient, based on a constellation of signs on multimodal imaging modalities such as fundus photo (FP), fluorescein angiography (FAG), and SD-OCT images. Patients who took tamoxifen or chloroquine derivatives, had diabetic retinopathy with 10 or more small retinal hemorrhages, and/or microaneurysms or other confounding retinal diseases were excluded. The collected images were retrospectively analyzed by a single retinal specialist (Y.H.K.).

The scanning methods of and characteristics observed on SD-OCT are described in detail in Report No. 1^6^. Briefly, the following models of SD-OCT were used in this study: 3D OCT-1000 Mark II, Cirrus HD-OCT (Models 4000 and 5000, Carl Zeiss Meditec, Dublin, CA, USA), Spectralis HRA + OCT (Heidelberg Engineering, Heidelberg, Germany), or DRI OCT Triton (Topcon Corp., Tokyo, Japan). The following characteristic findings on SD-OCT were recorded: disorganization of retinal inner layers, inner and outer retinal hyporeflective cavities, collapse of outer retinal layer, and disruption of the ELM, EZ, or IDZ. Disorganization of retinal inner layers was defined as boundaries between the ganglion cell-inner plexiform layer complex, inner nuclear layer, and outer plexiform layers that could not be identified and demarcated. Hyporeflective cavity was classified into “inner” and “outer” cavity based on the border between the inner nuclear land outer plexiform layers. At the foveolar center where is no inner retinal layers, the cavity located within the inner half of the total retinal thickness or in contact with inner retinal surface was defined as inner retinal hyporeflective cavity (Supplementary Figure S1).

Categories for disease progression suggested by Wong et al.^15^ was used to identify eyes in MacTel type 2 with severity 3. Eyes in severity 2 demonstrate normal lamination and lacked any foveal cystic or atrophic changes on OCT, while eyes in severity 3 demonstrate central outer retinal atrophy on OCT, either as intraretinal cystic degeneration or as central thinning with loss of outer retinal lamination^15^. Accordingly, eyes showing inner retinal cavities without outer retinal degeneration were classified as severity 3, rather than severity 2. Eyes diagnosed with MacTel type 2 with severity 3 were divided into two groups according to the presence of RAV on FP or FAG. RAV was defined as blunted, slightly dilated vessels that are mainly located in the temporal parafovea and that drive at a right angle into deeper retinal layers without narrowing towards the foveola^1,11^. Snellen visual acuity was converted to logMAR visual acuity for statistical analysis, and a generalized linear model with generalized estimation equations was used to account for the correlation between eyes in the same patient. Independent t-tests were used to compare continuous variables, while categorical variables were compared using a chi-square test or Fisher’s exact test. Logistic regression analysis was used to verify SD-OCT factors associated with RAV and presented as ORs and 95% CIs. All statistical analyses were performed using SPSS software version 25.0 (IBM Corp., Armonk, NY, USA). *P* values < 0.05 were considered statistically significant.

## Supplementary Information

Supplementary Information.
