# Supplementary material for: Characterizing right-angled vessel in macular telangiectasia type 2 with structural optical coherence tomography
Source: Sci Rep. 2021 Aug 25;11:17198. doi: 10.1038/s41598-021-96789-4 (PMC8387352; doi:10.1038/s41598-021-96789-4)
Supplement: Supplementary file 1 — Supplementary Information. [file 41598_2021_96789_MOESM1_ESM.pdf]

Characterizing right-angled vessel in macular telangiectasia type 2 with structural optical coherence tomography

Yoo-Ri Chung[1*], Young Ho Kim[2*], Jaeryung Oh[2], Seong-Woo Kim[2], Christopher Seungkyu Lee[3,4,5], Cheolmin Yun[2], Boram Lee[2], So Min Ahn[2], Eun Young Choi[3,4,5], Sungmin Jang[6], Kihwang Lee[1]

[1]Department of Ophthalmology, Ajou University School of Medicine, Suwon, Korea

[2]Department of Ophthalmology, Korea University College of Medicine, Seoul, Korea

[3]Department of Ophthalmology, [4]Institute of Vision Research, and [5]Institute of Human Barrier Research, Yonsei University College of Medicine, Seoul, Korea

[6]Retina Center, Saevit Eye Hospital, Goyang, Korea

[*]These authors contributed equally to this work

SUPPLEMENTAL INFORMATION

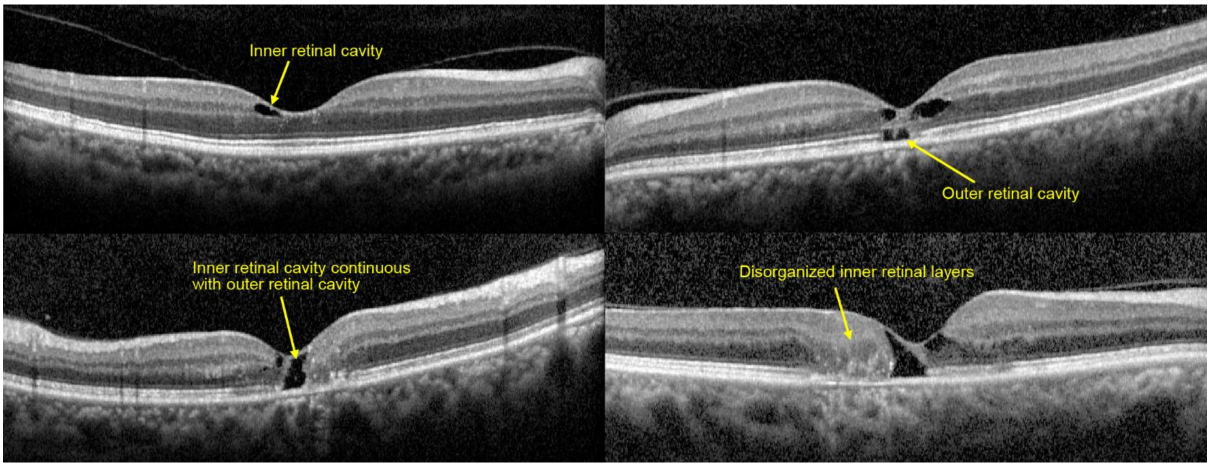

Figure S1. Various structural OCT findings in macular telangiectasia type 2.
